# Chronic activation of p38α in skeletal muscle causes necrotic changes, but also abolishes expression of MK2, MK3, and MKK6 and the muscle recovers

**DOI:** 10.1016/j.jbc.2026.111338

**Published:** 2026-03-04

**Authors:** Nechama Gilad, Ilona Darlyuk-Saadon, Manju Payini Mohanam, David Engelberg

**Affiliations:** 1 Department of Biological Chemistry, The Institute of Life Science, The Hebrew University of Jerusalem, Jerusalem, Israel; 2 Singapore-HUJ Alliance for Research and Enterprise, Molecular Mechanisms of Liver Inflammatory Diseases (MLID) Program, National University of Singapore, Singapore; 3 Department of Microbiology and Immunology, Yong Loo Lin School of Medicine, National University of Singapore, Singapore

**Keywords:** p38α, MAPK, MK2, MK3, MKK6, skeletal muscle, C2C12 cells

## Abstract

The MAPK p38α is associated with skeletal muscle’s development, differentiation and functionality. But, as it is overactive in muscle diseases and aging, it was proposed to be a pivotal promoter of these processes as well. It is not clear how p38α is involved in these disparate activities, in particular whether its chronic activation alone is sufficient to cause them. We established a mouse model designed to study the effects of p38α *per se* in skeletal muscle. p38α activation is achieved by inducible expression, in muscle, of an intrinsically active variant, p38α^D176A+F327S^. Two weeks following expression muscle degeneration and necrotic changes were observed, accompanied with elevation of p53, caspase 3, and γH2AX; and, intriguingly, suppression of the p38’s substrates MK2 and MK3 and its activator MKK6. At later timepoints the tissue recovered, apoptotic markers disappeared, but MK2, MK3, and MKK6 remained suppressed, perhaps as a response that restrains p38α-mediated damage and allows recovery. Induction of p38α^D176A+F327S^ in young mice (2 months old) caused milder effects, but MK2, MK3, and MKK6 were suppressed. The p38α^D176A+F327S^ effects were associated with altered level of ∼2000 mRNA molecules. For 1700 genes, the effect was transient and for ∼300 constant. Stress-induced activation of p38α in C2C12 myoblasts was also associated with MK2 downregulation, but with constant elevation of apoptotic markers. Thus, chronic activation of p38α *per se* in skeletal muscle is sufficient to cause damage reminiscent of aging effects, but cannot impose full-scale and lasting aging phenotype. The tissue recovers while suppressing the p38α pathway.

The mitogen-activated protein kinase p38α is a key element in the regulation of numerous physiological processes, including proliferation, differentiation, apoptosis, and survival. It is expressed in all eukaryotic cells and is essential for embryonic development (1, 2, 3, 4, 5, 6, 7). p38α is tightly regulated, being catalytically inactive unless dually phosphorylated on a unique TGY motif located within its activation loop (1, 2, 3, 8). TGY phosphorylation is commonly catalyzed by the MAPK kinases MKK6 and MKK3 (1, 2, 3, 9). Once activated, p38α phosphorylates a wide range of substrates, including transcription factors, chromatin-remodeling regulators, translational machinery components, and cytoskeletal proteins (1, 2, 9, 10). Important among the substrates are protein kinases known as MAPK-activated protein kinase 2 (MAPKAPK2) and MAPKAPK3 (MK2 and MK3), through which p38α activates inflammatory responses and, at times, various morbidities (2, 11, 12).

p38α was shown to be important for many aspects of the development, differentiation, functionality, and metabolism of skeletal muscle (13, 14, 15, 16, 17, 18, 19, 20). But, intriguingly, it is also associated with muscle degradation in response to injury (atrophy) or to aging (sarcopenia) (21, 22, 23). p38α is also associated with morbidities of the muscle, such as muscular dystrophy diseases, including Duchenne’s (24, 25, 26) and facioscapulohumeral muscular dystrophy (27, 28). p38 activity was reported to be elevated in these maladies and to enhance dystrophies by activating apoptotic cell death machinery (25, 26).

In the developing embryo, p38α is activated/phosphorylated in the early myotome (17, 29), suggesting that it plays a role in muscle development. It was also proposed to be critical for differentiation of myoblasts to multinuclear fibers in culture (in C2C12 and L8 cells) (15, 19, 20, 30, 31). Interestingly, however, in C2C12 cells, two other isoforms of the p38 family, p38β and p38γ, seem to be able to replace p38α when inactive (32).

p38 signaling was found to be involved in activating asymmetric division and differentiation of quiescence muscle stem cells (MuSCs; also known as satellite cells) to provide myoblasts for muscle repair (33, 34). Indeed, in mice conditionally knocked out for p38α in MuSCs, complete regeneration following injury is delayed (but not abolished) (16). These mice also manifest a modest reduction in muscle mass, caused by lack of postnatal hypertrophic growth, an effect that results from high activity of p38γ in these muscles and not of the lack of p38α (16).

Thus, p38α seems to be important for muscle development, differentiation, and repair/regeneration. But, confusingly, at the same time, it is also associated with atrophy and sarcopenia, and other symptoms of muscle aging (35, 36, 37, 38). Indeed, although aging-associated defects in MuSCs are believed to be consequences of multifactorial changes, including elevation of the Janus kinases-signal transducer and activator of transcription and the TGFβ pathways, elevated p38α activity in aged individuals is proposed to be a pivotal element in sarcopenia. This notion is strongly supported by the observation that exposure of MuSCs from old mice to p38α/β inhibitors rejuvenates them and improves their capability to differentiate to myoblasts (21, 23). Along this line, p38α activity was also proposed to be critical for denervation-associated atrophy, based on the observation that sciatic nerve denervation caused p38α activation; and p38α downregulation reduced atrophy in this experimental setup (39). Furthermore, in mice expressing an inactive allele of p38α, muscle denervation-induced atrophy was significantly less severe (40). These observations strongly relate p38α to atrophy induced by injury or aging. Yet, in some muscles p38α activity is high and constant from an early age (41), adding to the confusion regarding the function of p38α in skeletal muscle.

Most studies of p38α in muscle were based on monitoring p38α phosphorylation status under various conditions, and on experimental manipulations that reduced p38α activity through the use of pharmacological inhibitors, siRNA, or conditional knockouts (13, 15, 16, 19). The effect of p38α activation on the muscle has not been determined as it was not possible to impose its activation *per se in vivo*, let alone in a tissue-specific manner.

The p38α′s direct and prominent substrate MAPKAPK2 (MK2) was also shown to control both myogenesis, and, under different conditions (injury, aging), hypertrophy and atrophy (36). MK2 was also shown to protect muscle from atrophy *via* phosphorylation of Hsp27, which is a negative regulator of NF-κB in skeletal muscle, and inhibition of the ubiquitin E3 ligases MuRF1 and atrogin-1, preventing protein degradation (42, 43). These observations may imply that many of the p38α effects in skeletal muscle are mediated by activation of MK2 and perhaps also *via* its isoform MK3. p38α and MK2 reside in a protein complex in which they stabilize each other, so that if one protein is missing the other is downregulated (11, 44). But MK2 is also downregulated, *via* the E3 ligase MDM2 and the proteasome machinery, upon exposure of cells in culture to stresses that chronically activate p38. This stress-induced p38α activation and MK2 downregulation is associated with apoptosis (45). Some degree of downregulation of MK2 levels were also observed in tissues of transgenic mice expressing an intrinsically active variant of p38α (46). The effect of chronic p38α activity on MK2 in skeletal muscle *in vivo*, or in myoblasts in culture, is not known.

As studying the role of p38α in muscle by learning the effects of its activation is an approach that has not been taken, we established a mouse model for this purpose. In this model, p38α is activated in skeletal muscle by induced expression of the intrinsically active variant p38α^D176A+F327S^ (47, 48, 49). p38α^D176A+F327S^ spontaneously autophosphorylates/autoactivates itself on the TGY motif, independent of upstream signaling (50), and is therefore readily active upon expression. Below, we report that p38α activation *per se* in muscle causes necrotic changes, accompanied with modifications in the levels of more than 2000 genes. The effect is followed by regeneration and tissue recovery, and is more pronounced in 7 months old mice than in 2 months old mice. Intriguingly, p38α^D176A+F327S^ expression imposes a significant downregulation of MK2, MK3, pHsp27, MKK6, and endogenous p38α. We propose that elimination of these components is part of the tissue’s response that allows recovery under chronic p38α activity.

## Results

### Generation of a mouse model in which p38α activity *per se* is induced in skeletal muscle

To study the effects of p38α activation *per se* in skeletal muscle, *in vivo*, we sought a system that allows exclusive activation of this kinase in this tissue in a temporally controlled manner. It was thus tested whether the recently described p38α^D176A+F327S^-carrier mouse (51) could be suitable for this purpose. These mice allow tissue-specific and temporally controlled expression of the intrinsically active p38α^D176A+F327S^ molecule (a detailed description of the system is provided in ref. (51) and in Figure S1, that is adopted from reference (51)).

**Figure 1:**
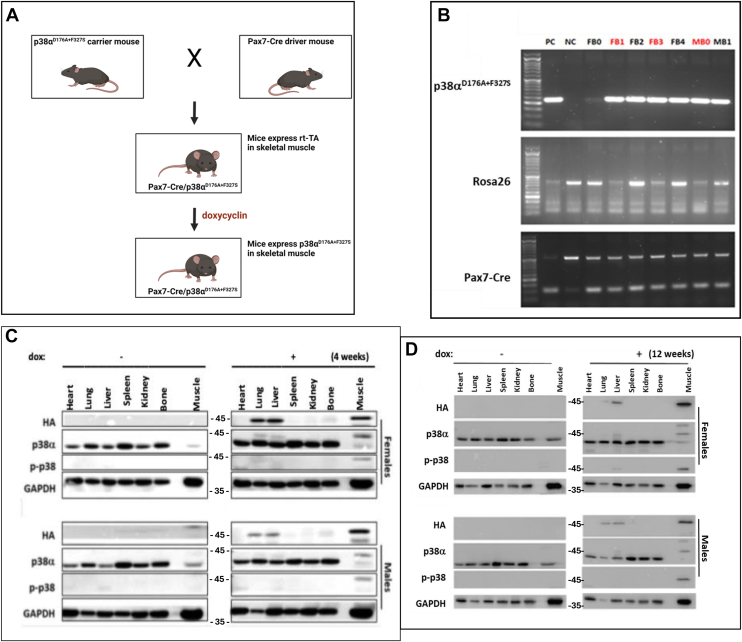
**Induction of p38α^D176A+F327S^ in Pax7-Cre/p38α^D176A+F327S^ mice is tightly controlled by doxycycline and the protein is activated/phosphorylated**. *A*, the specific mouse model established in this study. Pax7-Cre/p38α^D176A+F327S^ heterozygous (*middle panel*), are progeny of mating between the “driver” mice that harbor the Pax7-Cre transgene and the “carrier” mice that harbor the rtTA and the p38α^D176A+F327S^ expression cassettes described in the text and in Figure S1. *B*, a representative PCR results of genotyping the progeny from a cross breeding of Pax7-Cre mice with p38α^D176A+F327S^-carrier mice. For p38α^D176A+F327S^: Mice FB1, FB3, and MB0 are homozygous for the p38α^D176A+F327S^ transgene (positive for p38α^D176A+F327S^ and negative for Rosa26 locus). Mouse FB2, FB4, and MB1 are heterozygote (positive for p38α^D176A+F327S^ and positive for Rosa26 locus). FB0 is a WT (negative for p38α^D176A+F327S^ and positive for Rosa26 locus). For Pax7-Cre: *lower band* is positive, *upper band* is negative. All mice are heterozygote to Pax7-Cre. *C* and *D*, Western blot analysis, with the indicated antibodies, of protein lysates prepared from the indicated organs of Pax7-Cre/p38α^D176A+F327S^ mice, females (*upper panels*) and males (*lower panels*), fed with regular (*left panels*, (−)) or dox-supplemented (*right panels*, (+)) diets for 4 (*panel C*) and 12 (*panel D*) weeks. Note that the anti-HA antibodies react with the HA-p38α^D176A+F327S^ protein. Dox, doxycycline; FB, female black; MB, male black; PC, positive ctrl; NC, negative ctrl; Pax7, paired box protein 7.

To test whether this system could be exploited for establishing mice that inducibly express p38α^D176A+F327S^ in skeletal muscle, p38α^D176A+F327S^-carrier mice were crossed with paired box protein 7 (Pax7)-Cre driver mice (Fig. 1*A*). In these mice, transcription of the Cre recombinase is promoted by the promoter of the gene encoding Pax7, which is active in MuSC (52). Since in the course of embryo development Pax7 is expressed already at the dermomyotome, the precursor of all myogenic cells, progeny of the breeding between the p38α^D176A+F327S^-carrier mice and the Pax7-Cre driver mice are expected to express the Cre protein and consequently the rtTA transcription factor in all myogenic cells in the body. These mice are termed Pax7-Cre/p38α^D176A+F327S^. Notably, while rtTA is expressed in all myogenic cells of these mice, it is dormant and would induce transcription of p38α^D176A+F327S^ only when associated with doxycycline (dox) (Fig. 1*A*).

### In the Pax7-Cre/p38α^D176A+F327S^ mice, expression and activity of p38α^D176A+F327S^ is tightly controlled by dox availability

Genotypes of the progeny of the mating between the p38α^D176A+F327S^-carrier mice and the Pax7-Cre driver mice were confirmed by PCR (Fig. 1*B*). Then, mice were provided with dox-supplemented diet, and groups of mice were sacrificed 4 and 12 weeks later. Heart, lung, liver, spleen, kidney, bone, and the tibialis anterior (TA) skeletal muscle were collected. No expression of the p38α^D176A+F327S^ transgene was observed in any tissue, including skeletal muscle, taken from Pax7-Cre/p38α^D176A+F327S^ mice that were fed with a regular diet (Fig. 1, *C* and *D*, left panels), suggesting no leakiness of the system. In tissues removed from similar mice that were fed with dox-supplemented diet, high level of the HA-p38α^D176A+F327S^ protein was observed in the TA muscle (see the two upper rows in the right panels of Fig. 1, *C* and *D*), showing that expression of the transgene is faithfully induced by the presence of dox and is stable for at least 12 weeks. In further experiments, it was confirmed that expression is stable for longer periods (Fig. 6, *E* and *F* show expression after 28 weeks). Notably, expression of p38α^D176A+F327S^ is not absolutely restricted to muscle and was also monitored in lungs and liver (two upper rows in right panels of Fig. 1, *C* and *D*; see Discussion).

We then tested whether the HA-p38α^D176A+F327S^ molecules expressed in the muscle are spontaneously active. As the constant intrinsic catalytic activity of p38α^D176A+F327S^ stems from its autophosphorylation/autoactivation capability, as shown *in vitro* (47, 48), the possibility remains that in the mice, phosphatases would abolish spontaneous p38α^D176A+F327S^ phosphorylation/activity (53, 54, 55). Western blot analysis with anti-phospho-p38 antibodies revealed, however, that p38α^D176A+F327S^ is phosphorylated in skeletal muscle, and therefore resides in its active conformation (third row in right panels of Fig. 1, *C* and *D*). Unlike the p38α^D176A+F327S^ molecules expressed in skeletal muscle, those monitored in lungs and liver seem to be less active (third row in right panels of Fig. 1, *C* and *D*). We do not understand the expression of the transgene in lung and liver, nor the fact that it is phosphorylated at lower levels in these tissues (see Discussion).

It is concluded that in the mouse model established here, expression of p38α^D176A+F327S^ is induced by dox-supplemented diet and is active and stable for long periods of time. p38α^D176A+F327S^ expression occurs mainly in skeletal muscle, but also to some degree in lung and liver, in which it is less active.

### Induction of p38α^D176A+F327S^ expression in skeletal muscle evoked rapid, but transient, muscle degenerative response, including necrotic changes and elevation of apoptotic markers

**Figure 2:**
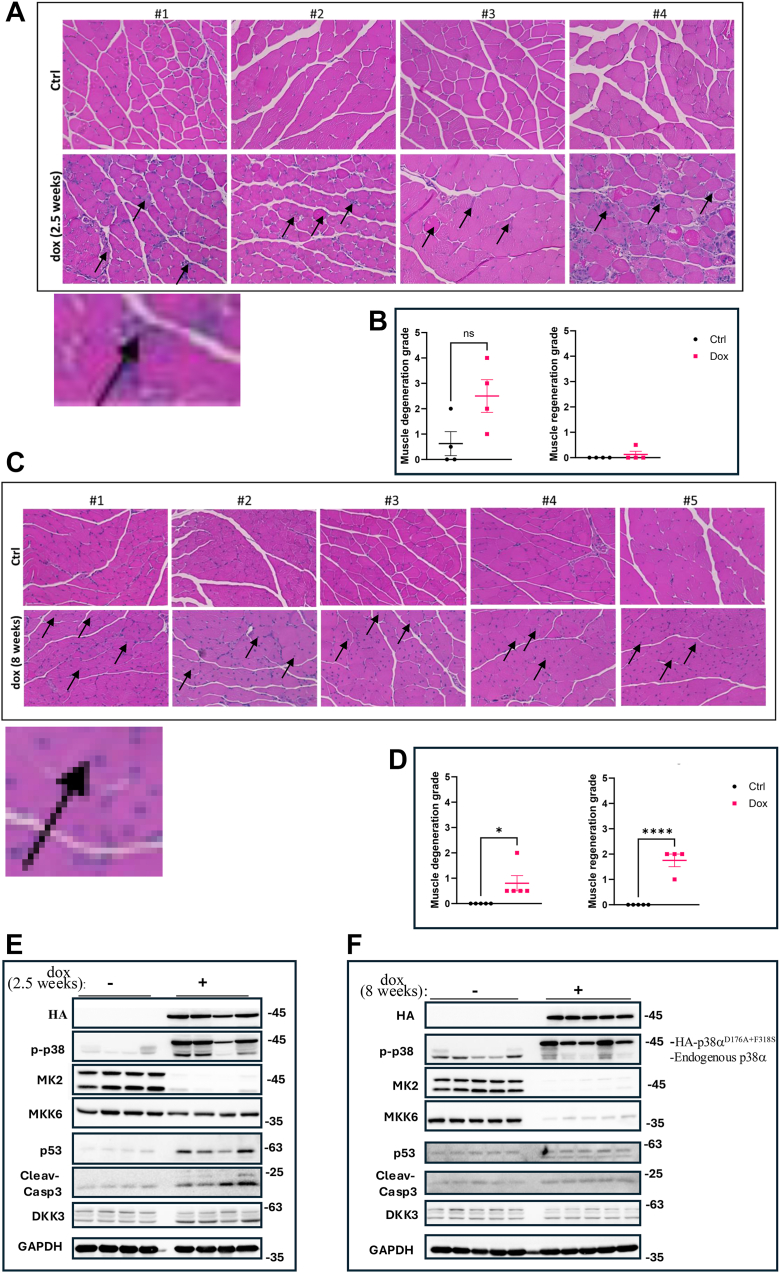
**Induction of p38α^D176A+F327S^ in Pax7-Cre/p38α^D176A+F327S^ mice resulted in rapid, but transient, muscle degeneration**. *A* and *C*, representative H&E staining of cross section of GC muscle from Pax7-Cre/p38α^D176A+F327S^ mice (7 months old) fed with a regular (Ctrl) or with dox-supplemented diets for 2.5 (*A*) and 8 weeks (*C*). *Arrows* in *panel A* point at degenerative changes, while *arrows* in *panel C* point at central nuclei. Insets show an enlarged region of each figure. *B* and *D*, pathological evaluation of degeneration (*left panels*) and regeneration (*right panels*) grade of muscle from Pax7-Cre/p38α^D176A+F327S^ mice treated with regular (Ctrl) or with dox supplemented diets for 2.5 (*B*) and 8 weeks (*D*) in muscle sections as presented in (*A*) and (*C*). *E* and *F*, Western blot analysis of lysates prepared from GC of Pax7-Cre/p38α^D176A+F327S^ mice fed with regular diet (−) or with dox-supplemented diet (+) for for 2.5 (*E*) and 8 weeks (*F*). Note that the anti-HA antibodies react with the HA-p38α^D176A+F327S^ protein. Dox, doxycycline; GC, gastrocnemius; Pax7, paired box protein 7.

Having confirmed operativity of the mouse model, we used it to ask whether induction of p38α^D176A+F327S^ in skeletal muscle might cause by itself symptoms commonly associated with aging. We thus provided dox-supplemented diet to 7-month-old Pax7-Cre/p38α^D176A+F327S^ mice and observed that after 2.5 weeks of p38α^D176A+F327S^ expression muscle manifested a marked degeneration and necrotic changes (Fig. 2, *A* and *B*). However, these effects did not last, so that 8 weeks after induction of p38α^D176A+F327S^ the degenerative grade decreased significantly, necrosis is almost not observed, and is replaced with massive regeneration, manifested by the appearance of central nuclei (Fig. 2, *C* and *D*; see arrows for central nuclei in Fig. 2*C*). Central nuclei are regeneration-related morphological markers as nuclei of cells of healthy muscle fibers reside in the periphery (56, 57, 58).

Testing markers that are associated with apoptosis and atrophy, such as Dkk3, p53, and cleaved caspase 3 (59, 60, 61, 62, 63, 64, 65), we observed increased levels at the 2.5-week time point (Fig. 2*E*), that were decreased at the 8-week time point (Fig. 2*F*). Namely, elevation of these markers is correlated with the observed degeneration and necrotic signs in the pathological analysis.

### Induction of p38α^D176A+F327S^ expression in skeletal muscle of young mice causes a transient regeneration-like process

**Figure 3:**
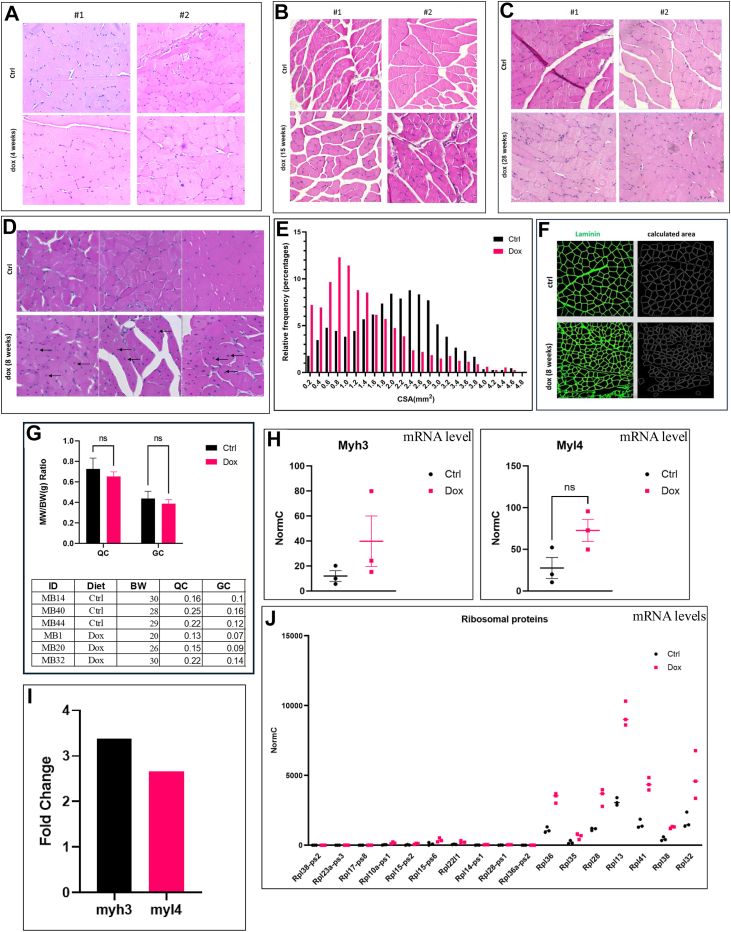
**Induced expression of p38α^D176A+F327S^ in young mice causes transient regeneration of skeletal muscle**. *A–D*, H&E staining of histological cross sections of QC (*A*) and GC (*B*–*D*) from Pax7-Cre/p38α^D176A+F327S^ homozygous mice fed with regular (control) or dox-supplemented (dox) diets for 4 (*A*), 15 (*B*), 28 (*C*), or 8 (*D*) weeks. Four mice, two from each experimental group, were tested at the 4, 15, and 28 weeks’ time points, and six mice were tested at the 8-week time point. *Arrows* in *panel D* point at central nuclei. *E*, relative frequency distribution of fibers’ of different calibers, as calculated by MuscleJ software (ImageJ), based on laminin immunohistochemistry (IHC) staining of cross sections of GC removed from mice fed with a regular diet (control) or a dox-supplemented diet is shown in *panel D* (n = 3 in each group). *F*, a representative image of laminin IHC staining of cross sections from mice fed with regular (Ctrl) or dox-supplemented diet (dox) for 8 weeks. *G*, muscle weight (MW) (QC and GC) to body weight (BW) ratio of mice that were fed with regular (Ctrl) or dox-supplemented (dox) diets for 2 months ((MW(g)/BW(g)) ∗100) (n = 3). *H*, normal counts of myh3 and myl4 mRNA levels from mice fed with a dox-supplemented diet, at the indicated time points following provision of dox, as revealed *via* RNA-seq analysis (two mice were tested at each time point). *I*, fold change of myh3 and myl4 mRNA levels in mice fed with dox-supplemented diet for 2 months compared to the levels in similar mice provided with regular diet as analyzed by RNA-seq (n = 3). *J*, normal counts of ribosomal proteins mRNA levels in mice fed with a dox-supplemented diet for 2 months compared to the levels in similar mice provided with a regular diet as analyzed by RNA-seq (n = 3). For more details regarding statistical analysis, see Materials and Methods. Dox, doxycycline; CSA, cross section area; GC, gastrocnemius; Pax7, paired box protein 7; QC, quadriceps; Myh3, myosin heavy chain 3; Myl4, myosin light chain 4.

We next tested whether induced p38α^D176A+F327S^ expression in animals as young as 2 months old will also cause aging-like symptoms. Quadriceps and gastrocnemius (GC) were collected from two groups of 2 months old Pax7-Cre/p38α^D176A+F327S^ mice, one provided with a regular diet and the other with a dox-supplemented diet for 4, 8, 15, and 28 weeks. H&E staining of cross sections revealed that fibers taken from mice provided with dox-supplemented diet for 4, 15, and 28 weeks were intact, similar to muscles of control mice (Fig. 3, *A*, *B* and *C*). However, in muscles taken from mice that were on dox-supplemented diet for 8 weeks, numerous cells with central nuclei were observed (Fig. 3*D*). Furthermore, p38α^D176A+F327S^ expression also affected the caliber of the fibers’ cross sections (Fig. 3, *E* and *F*) (a decrease in fibers’ caliber is another regeneration-related morphological marker (57, 58, 66)). Specifically, at the 8-week time point, muscles of p38α^D176A+F327S^-expressing mice were composed of significantly more fibers with smaller calibers' area as compared with control mice (Fig. 3, *E* and *F*). Total area of cross sections of muscles expressing or not expressing p38α^D176A+F327S^ was similar. Ratio of muscle weight to body weight was similar too (Fig. 3*G*).

Other known regeneration markers are elevated levels of myosin heavy chain 3 (Myh3), myosin light chain 4 (myl4), and ribosomal proteins. Myh3 and myl4 are commonly expressed transiently during muscle development in the embryo and during regeneration. In mature myofibers, they are substituted with adult's isoforms (67, 68). Ribosomal proteins are expressed constitutively, but during the regeneration process, in which the cells require high yield of proteins, their expression level increase. In Pax7-Cre/p38α^D176A+F327S^ mice provided with dox-supplemented diet, mRNA levels of Myh3 and myl4 and ribosomal proteins are elevated (Fig. 3, *H*, *I* and *J*), providing another indication that a regeneration process occurs in these muscles.

In summary, prompt activation of p38α *per se* in skeletal muscle (by inducing p38α^D176A+F327S^ expression) is sufficient to cause some atrophic/apoptotic symptoms, which are milder in very young mice and more severe in older mice. In both young and older mice, the effects are transient, suggesting that the muscle accomplishes a full recovery even though p38α^D176A+F327S^ is still expressed.

### Induction of p38α^D176A+F327S^ causes both transient and permanent changes in the steady-state levels of more than 2000 mRNA molecules

**Figure 4:**
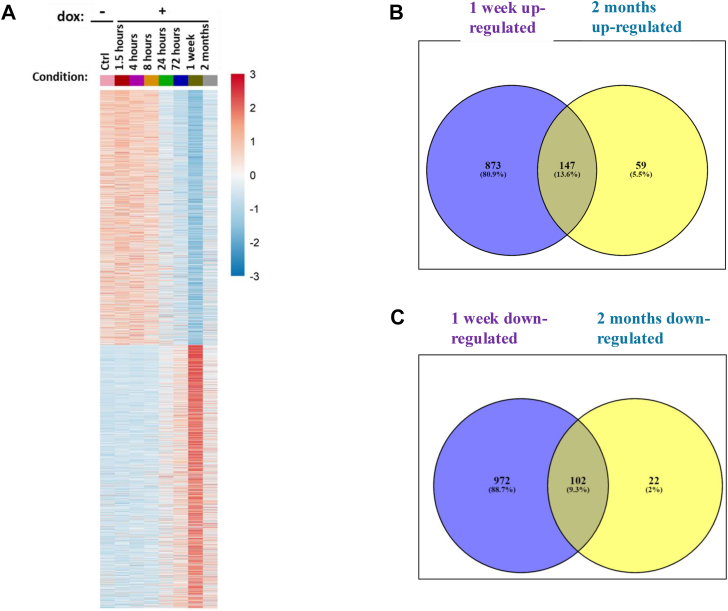
**Induction of p38α^D176A+F327S^ expression in skeletal muscle affects expression of more than 2000 genes**. *A*, heat map showing all genes affected by at least 2-fold following induction of p38α^D176A+F327S^ compared to mice provided with regular diet, in GC samples prepared from mice fed with dox-supplemented diet for the indicated periods, as determined by RNA-seq. Results at each time point are the averages of two experimental repeats. *B–C*, Venn diagrams of numbers of genes that were upregulated (*B*) or downregulated (*C*) after 1 week (*purple*) and 2 months (*yellow*). Dox, doxycycline; GC, gastrocnemius.

To look for the effect of p38α^D176A+F327S^ on the repertoire and levels of skeletal muscle mRNA population, RNA was extracted from GC samples of Pax7-Cre/p38α^D176A+F327S^ mice (2 months old at the beginning of the experiment) that were fed with dox-supplemented diet for 0, 1.5, 4, 8, 24, and 72 h, 1 week, and 2 months, and analyzed by RNA-seq. It was found that induction of p38α^D176A+F327S^ affected a large number of mRNA molecules, in various ways. A most dramatic effect was apparent 1 week after provision of dox-supplemented diet (Fig. 4*A*). At this time point, the levels of 2094 mRNA molecules were significantly different (*p* < 0.01) from their levels in control mice (not expressing p38α^D176A+F327S^) or from levels prior to induction of p38α^D176A+F327S^. One thousand twenty of these mRNAs were upregulated (Fig. 4*B*) and 1074 were downregulated (Fig. 4*C*), at least 2-fold. Similar to the necrotic changes and regeneration induced by p38α^D176A+F327S^, the dramatic effect on gene expression was mostly transient, as the levels of 873 of the 1020 genes that were upregulated (85.5%) and 972 of the 1074 genes that were downregulated (90.4%) at the 1 week time point returned to close to their original levels at the 2-month time point (Fig. 4, *A*, *B* and *C*). Importantly, however, part of the effect of p38α^D176A+F327S^ is long term, as 206 genes remained significantly upregulated and 124 genes remained downregulated at the 2-month time point (Fig. 4, *A*, *B* and *C*). Thus, although the tissue responded to the damage and seems to have reversed most of the changes imposed by p38α^D176A+F327S^, some of the effects caused by p38α^D176A+F327S^ were not reversed.

**Figure 5:**
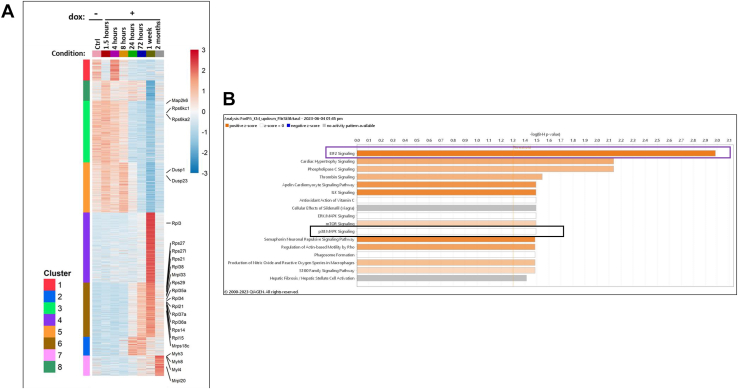
**Genes affected by p38**α**^D176A+F327S^ can be divided to 8 clusters.***A*, heat map of eight different clusters to which affected mRNA molecules were grouped according to common kinetics of the change in expression levels (some genes of interest are marked). *B*, a list of signaling pathways revealed by enrichment analysis of genes that were up or downregulated after the 2 months of p38α^D176A+F327S^ expression, based on RNA-seq analysis (2 independent experiments of 2 months treatment n = 3 and n = 2). [ Z-score, calculated by the IPA algorithm, predicts the activation or inhibition state based on the expression data] the bars' length corresponds to −log(BH *p* value), hence the statistical significance of the enrichment. The color of the bars represents the activation Z-score. *Orange*: activation, *blue*: inhibition. *White/gray*: not enough information to predict activation Z-score.

On the basis of the kinetics of their expression, the genes affected by p38α^D176A+F327S^ were grouped in eight distinct clusters (Fig. 5*A*; the complete list of genes in each cluster is provided in Supplementary file S1). Clusters 1, 3, 5, 6, and 7 contain genes whose levels remained significantly modified after 2 months of p38α^D176A+F327S^ expression (Fig. 5*A*). To assess whether the affected mRNAs may disclose biochemical pathways and/or physiological systems modified by the active p38α, an enrichment analysis was performed for the RNA-seq results (Fig. 5*B*; Supplementary file S2). This analysis revealed that 2 months of p38α^D176A+F327S^ expression induced changes in genes encoding components of the “eIF2 signaling pathway” (Z-score 2.33) that is involved in regulating protein synthesis and includes ribosomal proteins of both the small and the large subunits, indicating an increase in protein translation (Fig. 3, *H*–*J*). Genes encoding proteins that function in the so called “cardiac hypertrophy pathway” also manifested significant changes (Z-score 1.5) (Fig. 5*E*), with increase in expression of mRNAs encoding several myosin light chain proteins. Changes in the myosin pattern is one of the markers for regeneration as during this process embryonic/fetal myosins are expressed and switched to adult myosins at the end of the process. The enrichment analysis further presents changes in mRNAs encoding p38α pathway components and includes downregulation of mRNA encoding MKK6 (map2k6) (Fig. S5, *A* and *B*).

### Expression of p38α^D176A+F327S^ causes dramatic and permanent downregulation of components of the p38α pathway

**Figure 6:**
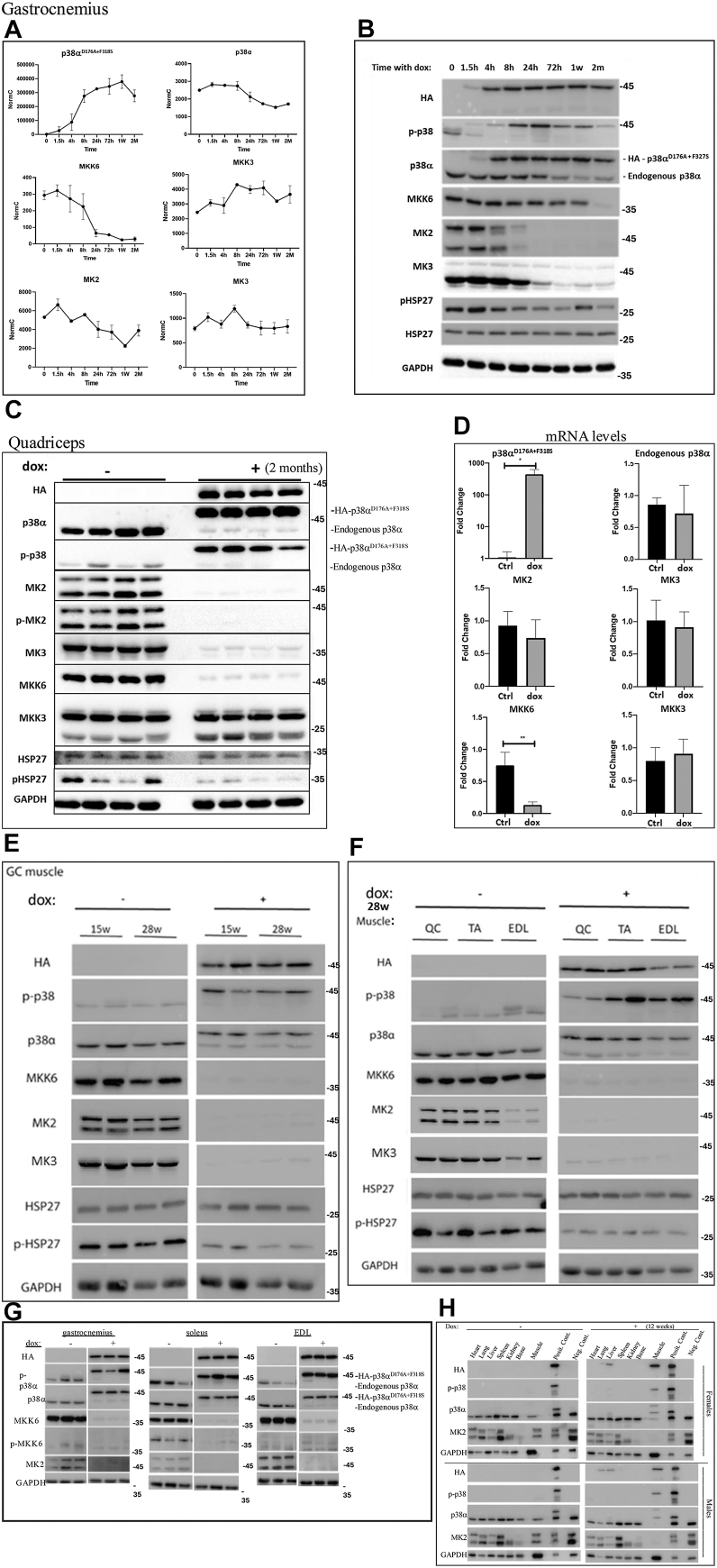
**Induced expression of p38α^D176A+F327S^ in skeletal muscle causes dramatic downregulation of upstream and downstream components of the p38α pathway**. *A*, normal counts of mRNA levels of the indicated genes as analyzed by RNA-seq of total RNA extracted from GC of Pax7-Cre/p38α^D176A+F327S^ homozygous mice that were fed with dox-supplemented diet for the indicated time periods (N = 2 at each time point). *B*, Western blot analysis, with the indicated antibodies, of protein lysates prepared from the same muscle of the same mice from which the RNA was isolated (shown in *panel A*). *C* and *D*, Western blot (*C*) and qRT-PCR (*D*) analyses of protein lysates or total RNA prepared from QC muscle of Pax7-Cre/p38α^D176A+F327S^ heterozygous mice that were not provided (−) or provided (+) with dox-supplemented diet for 2 months [regular diet = Ctrl; dox-supplemented diet = dox. (n = 4 at each time point)] [fold change = ratio of expression]. *E–G*, Western blot analysis of lysates prepared from QC, TA, EDL (E), and GC + soleus (*F*), or GC, soleus, or EDL (*G*) muscles of Pax7-Cre/p38α^D176A+F327S^ homozygous mice, 15 and 28 weeks (*E* and *F*), or 2 months (*G*) after providing of dox-supplemented (+) or regular (−) diets. Samples were taken from two (a male and a female) or three mice at each condition and were tested with the indicated antibodies. Differences in the levels of pHsp27 in *panel F* are attributed to the difference between the male and the female mice. *H*, MK2 downregulation in Pax7-Cre/p38α^D176A+F327S^ provided with dox-supplemented diet is specific to muscle. Lysates of the indicated tissues, collected from homozygous mice (same samples used in Fig. 1, *C* and *D*), were assayed *via* Western blot analysis probed with antibodies specific to the indicated proteins. Note that with the exception of the MK2, S-MyHC, and F-MyHC rows, other rows are identical to those in Fig. 1, *C* and *D*. Also note that in *panels B*, *C*, *and E–H* the anti-HA antibodies react with the HA-p38α^D176A+F327S^ protein. Dox, doxycycline; EDL, extensor digitorum longus; GC, gastrocnemius; Pax7, paired box protein 7; qRT-PCR, quantitative real-time PCR; QC, quadriceps.

Cluster 3, composed of genes that remain downregulated after 2 months of p38α^D176A+F327S^ expression, includes the mRNA encoding the Map2K MKK6, the direct activator of p38α (marked in Fig. 5*A*). We verified this finding by direct quantitative real-time PCR and found that MKK6 mRNA is downregulated significantly from the 8-h time point onward (Fig. 6*A*, left middle panel). To test whether downregulation of MKK6 mRNA is reflected at the protein level, a Western blot analysis was performed on protein lysates prepared from the same muscles used for the RNA-seq study. It was revealed that MKK6 protein levels were indeed significantly downregulated, but at a different kinetic than that of the mRNA levels (Fig. 6*B*, fourth row). Namely, while mRNA levels declined within 24 h after initiation of p38α^D176A+F327S^ expression, protein levels were significantly reduced after 2 months. Notably, expression and activity of p38α^D176A+F327S^ are already apparent at high levels 4 h after provision of dox-supplemented diet and remained stable for the 2 months of the experiment (Fig. 6*B*, first row).

To check if among components of the p38 pathway downregulation is unique to MKK6, we further monitored, on the same samples, the status of the other p38 activator MKK3 and of the p38α substrates MK2 and MK3. The MK2 and MK3 proteins, which were expected to be strongly phosphorylated following p38α^D176A+F327S^ induction, were rather dramatically and rapidly downregulated (Fig. 6*B*). They began to decline about 8 h after p38α^D176A+F327S^ induction and remained at very low levels (MK2 was in fact barely detectable) at all later time points (Fig. 6*B*). This significant downregulation was apparent at the protein level, while the encoding mRNA molecules were not affected (Fig. 6*A*). Finally, in addition to causing downregulation of MK2, MK3, and MKK6, the active variant also caused abolishment of the low basal phosphorylation levels of endogenous p38α as well as expression of the protein, but with a slower kinetic (Fig. 6*B*). The levels of pHsp27, a substrate of MK2/3, were also downregulated (Fig. 6*B*).

The dramatic reduction in steady-state level of the pathway’s components raised a concern that it might be specific to the GC muscle and not general. Proteins and mRNAs were therefore extracted from the quadriceps muscle of Pax7-Cre/p38α^D176A+F327S^ mice that were provided with dox-supplemented diet for 2 months. In this muscle too, protein levels of MKK6, endogenous p38α, MK2, and MK3 were very low (Fig. 6*C*), while at the mRNA level, only that of MKK6 decreased significantly (Fig. 6*D*). MKK3 was not affected. We further analyzed the fast fiber type TA, and the mixed fiber type quadriceps and GC from mice fed with dox-supplemented diet for 15 and 28 weeks (Fig. 6, *E* and *F*), and soleus (slow type muscle), GC and extensor digitorum longus from mice treated with dox-supplemented diet for 2 months (Fig. 6*G*). In all muscles tested, expression of p38α^D176A+F327S^ resulted in downregulation of MKK6, MK2, MK3, and endogenous p38α (Fig. 6, *E*, *F* and *G*). The observation that the effect is present in all of the muscles that were tested raised the concern that downregulation of these proteins is a defect of the experimental model and may occur in all tissues in response to dox-supplemented diet, not necessarily associated with p38α^D176A+F327S^ expression. We thus checked, heart, lung, liver, spleen, kidney, and bone for the level of MK2 and found that it was not downregulated in these tissues, confirming that the effect is specific to skeletal muscle cells expressing p38α^D176A+F327S^ (Fig. 6*H*).

Thus, activation of p38α in skeletal muscle results in very low levels of upstream and downstream pathway’s components, significantly affecting the MKK6–p38α–MK2/MK3 axis in all muscles. This downregulation is maintained throughout the course of the experiments (up to at least 28 weeks) and occurs whether p38α^D176A+F327S^ is induced in young or older mice (for seven mounts old mice, see Fig. 6, *E* and *F*). Namely, the recovery of the muscle from the necrotic effects imposed by p38α^D176A+F327S^ occurs when MKK6, MK2, and MK3 levels are extremely low. Perhaps downregulation of these inflammation-promoting proteins is required for muscle recovery.

The mechanism underlying the downregulation is component-specific; MKK6 is downregulated at the mRNA level while MK2, MK3, and endogenous p38α are downregulated at the protein level.

### The effects of p38α^D176A+F327S^ on pathways’ components are readily reversible upon its suppression

**Figure 7:**
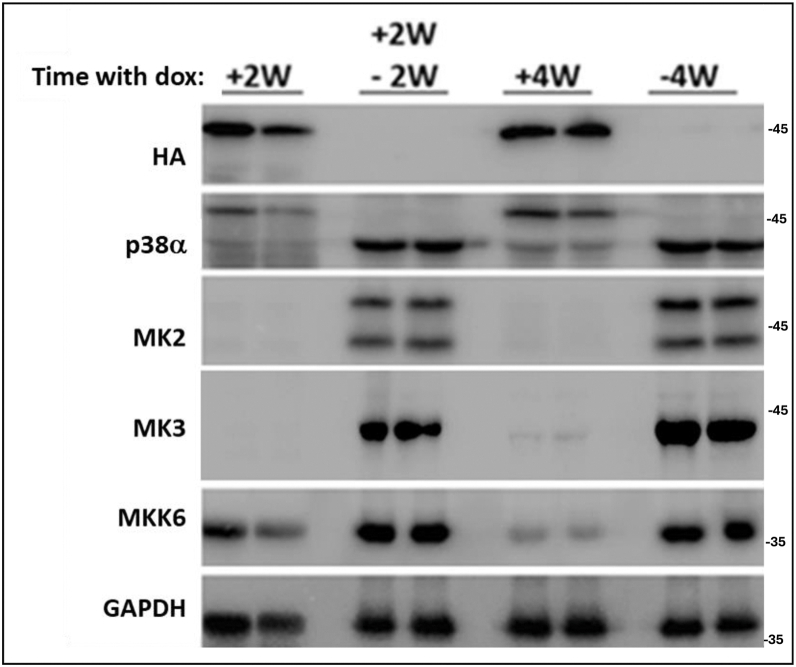
**Induction of p38α^D176A+F327S^ expression by dox-supplemented diet and its effect on components of the p38α pathway are fully reversible**. Lysates prepared from TA muscle of Pax7-Cre/p38α^D176A + F327S^ mice fed with a dox-supplemented diet for 2 weeks (+2W), a dox-supplemented diet for 2 weeks and then with a regular diet for another 2 weeks (+2W −2W), a dox-supplemented diet for 4 weeks (+4W), or a regular diet for 4 weeks (−4W), were analyzed by Western blot using the indicated antibodies. Dox, doxycycline; Pax7, paired box protein 7; TA, tibialis anterior.

To test whether p38α^D176A+F327S^ caused a permanent rewiring of signaling pathways in muscle cells so that the effect on MK2, MK3, and MKK6 is irreversible, or, perhaps, expression of these components could be de-suppressed, Pax7-Cre/p38α^D176A+F327S^ mice were fed with dox-supplemented diet for 2 weeks and then switched to a regular diet for additional 2 weeks. Western blot analysis of protein lysates prepared from the TA muscle showed that changing the dox-supplemented diet to a regular diet suppressed expression of the HA-p38α^D176A+F327S^ protein, manifesting the devotedness of the experimental model. In parallel, downregulation of the active variant readily reconstituted the expression of endogenous p38α, MK2, MK3, and MKK6 (Fig. 7). It seems that all effects on pathway’s components are directly dependent on p38α^D176A+F327S^ expression. Perhaps the pathway’s attenuation is a protective response of the tissue against the constant p38α activity, because uncontrolled MK2 and MK3 activity would cause chronic inflammation and associated muscle maladies (11, 12, 69, 70). Permanent downregulation of MK2/3 is probably the best alternative under the circumstances of chronic p38α activity, but is clearly a risky solution. Elimination (knockout) of MK2/3, at the whole animal level renders it sensitive to pathogens ((71); see also in Discussion). The effect of tissue-specific knockout of MK2/3 is not known, but may also render the tissue sensitive to pathogens hence the readily reversible character of the downregulation operated by the muscles in response to p38α^D176A+F327S^.

### Target genes of the p38α pathway are elevated in response to induction of HA-p38α^D176A+F327S^, suggesting that HA-p38α^D176A+F327S^ does not function as a dominant negative factor

**Figure 8:**
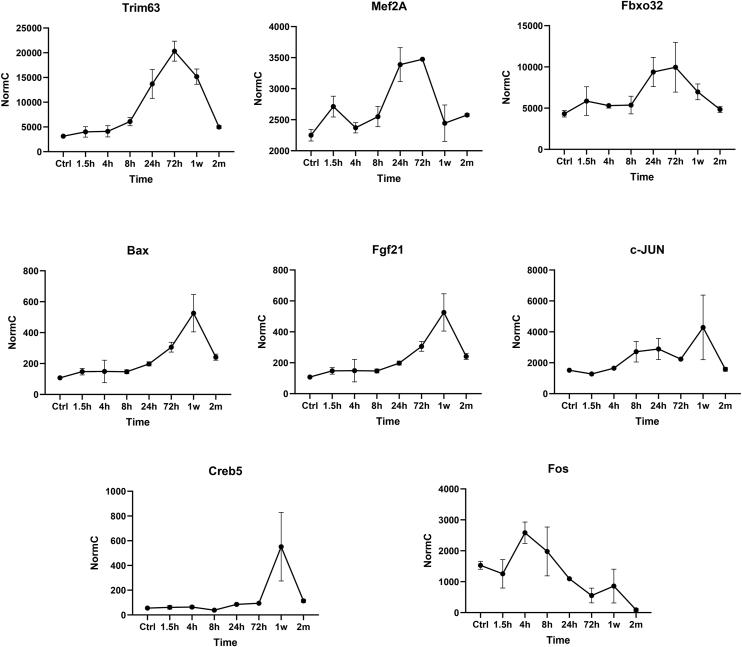
**Known target genes of the p38α pathway are induced in muscle upon induction of p38α^D176A+F327S^ expression**. Shown are normal counts of mRNA levels of the indicated genes as analyzed by RNA-seq of total RNA extracted from GC of Pax7-Cre/p38α^D176A+F327S^ homozygous mice that were fed with dox-supplemented diet for the indicated time periods (N = 2 at each time point). Dox, doxycycline; GC, gastrocnemius; Pax7, paired box protein 7.

The dramatic and permanent downregulation of components of the p38 pathway could be a result of a dominant-negative effect of the p38α^D176A+F327S^ on the p38 signaling pathway. To check for such a possibility, we monitored the levels of genes, whose induced expression was reported to be dependent on activation of the p38α pathway. These included the genes encoding Trim63/Murf-1 (72, 73, 74), Mef2A (75), Atrogin/FBXO32 (74, 76), Bax (77, 78), FGF21 (79), c-jun (80, 81), CREB5 (82) and FOS (83). The premise was that if p38α^D176A+F327S^ acts as a dominant negative factor, not only these genes would not be induced, but they might be downregulated. Of the sample of genes tested only FOS was downregulated (Fig. 8). mRNA level of Trim63, Mef2A, Fbox32, Bax, and FGF21 were high 1 week after induction of p38α^D176A+F327S^, long after MK2 and MK3 were significantly downregulated (8–24 h after induction; Fig. 6*B*). Although these observations cannot be taken as a conclusive proof that HA-p38α^D176A + F327S^ has no dominant negative effects, they do suggest that ultimate downstream targets of the cascade are activated by HA-p38α^D176A+F327S^. The fact that an upstream component, MKK6, is also downregulated (Fig. 6, *B* and *C* and *E–H*, and Fig. 7) also votes against the notion of a dominant negative effect of HA-p38α^D176A+F327S^. As p38α^D176A+F327S^ does not act as a dominant negative component on gene expression, perhaps the downregulation of MK2, MK3, MKK6, and endogenous p38 is not a consequence of a dominant negative effect either.

### p38α^D176A+F327S^-induced downregulation of MK2 in C2C12 myoblasts seems to be regulated by the proteasome and to be associated with apoptosis

Another potential mechanism that may explain the reduction in MK2/3, MKK6, and endogenous p38α levels is activation of a proteolytic machinery, specific to these molecules. This notion is based on a previous study that described MK2 downregulation *via* the ubiquitin-proteasome system in response to stress-induced p38α activity (45). This study described the phenomenon in cell cultures (U2OS cells, MEFs and CAFs) and showed that MK2 downregulation in these cultures leads to apoptosis (45). This effect somewhat resembles that of p38α activation in the 7 months old Pax7-Cre/p38α^D176A+F327S^ mice, as apoptotic markers were elevated 2.5 weeks after p38α^D176A+F327S^ was expressed (Fig. 2*E*). But unlike the case reported in cells in culture, in the mice these markers were not observed at later time points, even though MK2 was not re-expressed (Fig. 2*F*). Thus, *in vivo*, the tissue is capable of reversing initial induction of apoptotic signals so that the prolonged downregulation of MK2, MK3, and MKK6 is not associated with apoptosis, but rather with repair.

**Figure 9:**
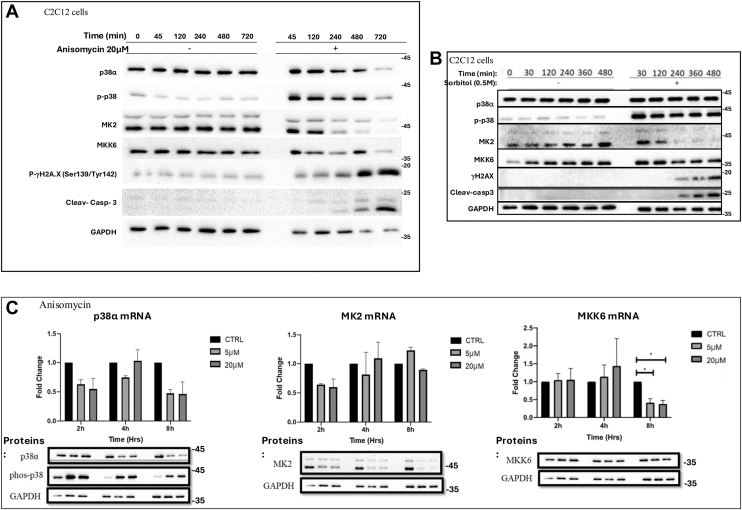
**In the myogenic cell line C2C12, stress-induced activation of p38**α **causes downregulation of MK2 and MKK6, and induction of apoptotic markers**. *A* and *B*, Western blot analyses of protein lysates prepared from C2C12 myoblasts exposed to anisomycin (*A*) or sorbitol (*B*) for the indicated period and analyzed with the indicated antibodies. *C*, downregulation of MKK6 in response to anisomycin is regulated at the mRNA level, while downregulation of p38α and MK2 occur at the protein level. C2C12 were exposed1 to 5 μM or 20 μM anisomycin for the indicated periods. Total RNA was isolated, and protein lysates were prepared and analyzed *via* qRT-PCR and Western blot respectively. Note that the GAPDH panels are the same in the three columns of *panel C* as all panels are showing results of the same experiment. qRT-PCR, quantitative real-time PCR.

**Figure 10:**
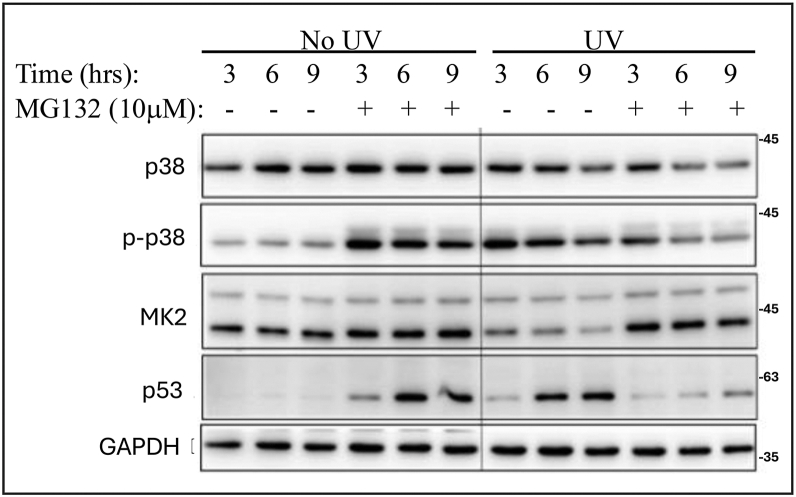
**Exposure of C2C12 cells to UV radiation causes p38α activation and MK2 downregulation that is prevented by a proteasome inhibitor**. Cells were exposed to UV-C light (2 J/m^2^/sec) for 15 s, in the presence or absence of MG132 and cell lysates were prepared at the indicated time points afterward. Lysates were analyzed by Western blots with the indicated antibodies.

This difference between the effect of stress-induced p38α activation followed by MK2 downregulation in cell lines (apoptosis), and that of p38α activation followed by MK2 downregulation in intact skeletal muscle (transient damage followed by recovery), raised the question whether myogenic cells in culture behave similar to the tissue or similar to (nonmyogenic) cell cultures. We thus exposed cells of the myogenic cell line C2C12 to anisomycin, sorbitol, or UV radiation and observed that these treatments led to p38α activation and MK2 downregulation (Figs. 9 and 10). The MKK6 protein was downregulated in response to anisomycin, but only 12 h after treatment and was not downregulated in response to sorbitol. MKK6 mRNA levels were downregulated, but not those of MK2 (Fig. 9*C*). In parallel, the apoptotic markers caspase 3 and phosphorylated histone H2A.X (at Ser139 and Tyr142) were significantly elevated (Fig. 9, *A* and *B*). Massive cell death was observed in response to all treatments. Cells detached from the plates and no recovery was monitored. We conclude that when p38α is induced in muscle cells in culture in response to stress, MK2 is downregulated, as observed for other cell types and in the intact tissue. But unlike the response of the whole animal, in culture the cells do not recover. Obviously, while in the Pax7-Cre/p38α^D176A+F327S^ mouse model p38α is induced individually, in culture cells exposed to anisomycin, sorbitol, or UV, p38α is coinduced with other stress responses that may interfere with recovery attempt. Also, these treatments cause a significant damage to the cell, that most probably do not occur when p38α^D176A+F327S^ expression is not accompanied by any other treatment (see Discussion).

As mentioned already, in previously tested cell lines, downregulation of MK2 in response to stresses that evoke p38α activation were reported to occur *via* the ubiquitin-proteasome system (45). To assess whether in myogenic cells too MK2 might be regulated by this machinery, we exposed C2C12 cells to UV radiation in the presence or absence of the proteasome inhibitor MG132 (right panel in Fig. 10). UV radiation did cause a significant reduction in MK2 levels, but not as dramatic as observed in the mice. Nevertheless, when the myoblasts were exposed to UV radiation in the presence of 10 μM MG132 MK2 was not downregulated (Fig. 10, right panel, third row). It seems that similar to the observation with U2OS cells, MEFs and CAFs, also in C2C12, p38-dependent MK2 downregulation is executed by the proteasome.

## Discussion

As the role of p38α in skeletal muscle is complex and ambiguous, novel approaches should be taken to assist in deciphering it. The Pax7-Cre/p38α^D176A+F327S^ mouse model, in which the effect of p38α on skeletal muscle is studied by activating it rather than by its inhibition or downregulation, constitutes such an approach. As active p38α can be readily induced, the model would be beneficial for learning the effects of p38α on skeletal muscle at various stages of the animal life and under different conditions, for example, in the course of embryonic development, recovery from injury, physical activity, aging, denervation, and development of muscle diseases. Also, the p38α^D176A+F327S^-carrier mouse model is constructed in such a way that p38α^D176A+F327S^ could be expressed in any cell type in a specific manner, including, for example, in muscle fibers, or in cardiac muscle (by the use of a suitable driver mouse). The model is based on the premise that once p38α^D176A+F327S^ is expressed in a given tissue it executes the very same functions that native p38α executes when activated by MKK6 or MKK3. It must be taken into consideration, however, that p38α^D176A+F327S^ is autoactivated by autophosphorylation, bestowed on the enzyme by the mutations, a mechanism of activation that may confer on it properties that are not identical to those of natively activated p38α. Yet, our analyses of the p38α^D176A+F327S^ molecule suggest that it does share many properties with the natively activated p38α and should be legitimate for physiological studies (49, 50). In any case, to our knowledge there is currently no other way to activate p38α *per se in vivo*.

The current study took advantage of the Pax7-Cre/p38α^D176A+F327S^ mouse model to learn the effect of p38α activation *per se* on the myogenic system of adult mice without further manipulations. The major observation at the physiological level is that expression of the active p38α was sufficient by itself to cause damage, and that regeneration responses were induced and damage was repaired. The recovery process was operative although p38α activity remained elevated. It can be concluded that activation of p38α is not sufficient to cause by itself a lasting irreversible damage. It is plausible that to cause an irreversible damage or a degenerative disease p38α activation should be combined with other factor(s). Aging might be such a factor because when p38α^D176A+F327S^ is induced in older mice (in fact just 7 months old) damage was apparent earlier after induction, and lasted longer, than its effect on 2-month old mice.

In summary, p38α^D176A+F327S^ *per se* is capable to cause alterations in the tissue, which are less significant in young mice, but when it is the only relevant activated component, atrophy and other aging phenotypes do not further develop and the tissue recovers. This is probably because the molecular basis of long-term damages and muscle maladies, such as atrophy, sarcopenia, or dystrophies, is not a result of a single but of various biochemical activities, p38α activation being just one of them. This notion is exemplified by the observation that cells in culture undergo apoptosis in response to chronic p38α activation and MK2 downregulation (ref. (45); and Fig 9, *A* and *B*), probably because in response to stress p38α was not induced alone, but together with other stress- and damage-responsive cellular activities. Also, obviously, cells in culture are not associated with body systems, and under such situation the repair and recovery machinery are ailing. Perhaps *in vivo* too, the effect of p38α activation when occurs concomitantly with exposure to stress or injury, would be more severe, would last longer and, on occasion, would not be repairable.

While the effect of chronic p38α^D176A+F327S^ expression on muscle organization and structure is reversible, part of its effect on gene and protein expression, particularly on components of its own pathway, is permanent. Hence, the recovery and regeneration processes take place when major components of the p38α pathway remain at very low, almost undetectable, levels. It could be that downregulating MKK6, MK2, and MK3 is in fact required for the recovery, operating as a protective response from the potential deleterious effects of chronic activity of the p38α/MK2/MK3 pathway. If this is indeed the case, downregulation of pathway’s components is a solution for allowing recovery and functionality of the muscle in the presence of constitutively active p38α. The ability to employ such a mode of protecting the tissue seems important given that evolution equipped organisms with several mechanisms for this purpose; such as mRNA downregulation for MKK6 and protein degradation for MK2. This protective response seems legitimate and less hazardous to the tissues since abolishment of MK2 and MK3 is not immediately harmful. Mice knocked out for the genes encoding MK2 and MK3 manifest normal development and fertility, and are even more resistant then WT mice to inflammatory signals (11, 12). However, MK2^−/−^ and MK2/3^−/−^ mice are sensitive to pathogens (71). Extrapolating from these studies, it could be that when expressing p38α^D176A+F327S^ Pax7-Cre/p38α^D176A+F327S^ mice may encounter difficulties in coping with stresses, injuries, pathogens, denervation, or metabolic changes. Therefore, although MK2/MK3 downregulation may assist in recovery from constant p38α activity, it may also render the organism sensitive to stresses and infections. In the model used here, these sensitivities are expected to be specific to muscle, as MK2 and MK3 are normally expressed in other tissues when p38α is activated in muscle (Fig. 6*H*).

It could also be, on the other hand, that MK2/3 and MKK6 downregulation is not associated with recovery but rather with the necrotic effects. In such a model downregulation of these components is part of the pathology in diseases (see Discussion in (84)). To resolve these matters, it is important to monitor the status of MK2/3 and MKK6 in human chronic inflammatory diseases and in response to anti-inflammatory treatment (including inhibition of p38α). This would be informative for the design of a proper therapeutic strategy (inhibition or activation of MK2/3 for example; (84)). On the basis of the observation with the Pax7-Cre/p38α^D176A+F327S^ mice, our favorite model is that when p38 is chronically activated, due to constant stress, for example, the tissue protects itself by downregulating MK2/3 and MKK6. Diseases would develop in those individuals in which the protective machinery fails and the MK2/3 pathway is reactivated, remains chronic, and ignites inflammation. This idea may be more relevant to muscle than to other tissues, because of the dramatic degree of components downregulation. When p38α^D176A+F327S^ is expressed in all mouse tissues, MK2 downregulation was significant, but the protein was still detectable (46); and when p38α^D176A+F327S^ was expressed specifically in the liver there was an effect on MK2, but not as significant as in muscle (51). In mice expressing p38α^D176A+F327S^ in the lung, no downregulation of MK2 or MK3 was observed, perhaps because in the lung p38α^D176A + F327S^ did not cause a significant damage (85). In tissue culture as well, no total elimination of MK2 was observed (Figs. 9, *A* and *B*, 10 and ref. (45)). It is not clear to us why the negative feedback in skeletal muscle is more efficient than in other tissues.

It is not known what triggers constant activity of p38α in chronic inflammatory diseases and aging. The trigger may be disease-specific and tissue-specific, given that aging is asynchronous between tissues, even between muscles (41), and given the diversity of chronic inflammatory diseases. Current understanding of the matter does not consider mutations to be part of the trigger (unlike the case of cancer). As mutated, intrinsically active p38α supports muscle necrosis, and when expressed in liver causes symptoms of fatty liver disease (51), it would be interesting to look for mutations in the p38α pathway in patients suffering of chronic inflammation.

The dramatic downregulation of MK2, MK3, MKK6, and endogenous p38α, is an extreme case of negative feedback. Negative feedback activities play critical roles in controlling major processes of life (86, 87, 88). In signal transduction cascades, negative feedbacks seem to be particularly critical as exemplified in the proto-oncogenic receptor tyrosine kinase–Ras–Raf–MEK–Erk pathway (89, 90, 91, 92, 93). Understanding the feedback machinery is important because when a targeted therapy drug intercepts its target, it blocks the target’s downstream pathological effects, but at the same time abolishes its negative feedback effects, causing overactivation of the cascade (90, 91, 92, 93).

Finally, we want to point out that in the Pax7-Cre/p38α^D176A+F327S^ mice expression of p38α^D176A+F327S^ is induced in cells that express Pax7 (as Pax7-cre driver mice were used) and their progeny. In the course of embryonic development, Pax7 is expressed in MuSCs that later differentiate into myoblasts and ultimately to myofibers so that p38α^D176A+F327S^ is expressed in all cell types of the myogenic system, including satellites, myoblasts, and mature fibers. But, as Pax7 is expressed in the mesodermal layer of the early developing mouse embryo, and is known to be expressed in some neuronal cells (94, 95, 96), p38α^D176A+F327S^ is expressed in some other tissues, in addition to skeletal muscle.

## Experimental procedures

### Mice

#### Establishment of Pax7-Cre/p38α^D176A+F327S^ transgenic mice

Mice were housed in ventilated cages with a constant temperature of 25 °C, 30 to 70% humidity, 12-h light/dark cycle. The protocols for mouse breeding BR20-0795 and research R20-0799 were approved by the IACUC, NUS, and breeding and research protocol NS-19-15807-4 by the IACUC, HUJI.

The p38α^D176A+F327S^-carrier transgenic mice (see details in refs. (46, 51)) were cross bred with the driver mice Pax7-Cre (Pax7tm1(Cre)Mrc/J. Strain #:010530, Jackson laboratory). The progeny of this mating harbor one copy of the p38α cassette and one copy of the Pax-Cre cassette, as was verified by genomic PCR, and were termed Pax7-Cre/p38α^D176A+F327S^ heterozygous. To obtain mice homozygous for the transgenes, heterozygous were further crossed between themselves and homozygous were identified by genotyping.

##### Mouse genotyping

Genomic DNA was extracted from mice tails and amplified by REDExtract-N-Amp Tissue PCR Kit (XNAT-100RXN; Sigma-Aldrich) following the manufacturer’s protocol. PCR primers and the amplicon sizes are shown in Table 1.

**Table 1:** Primer used for genotyping and expected amplicon size

Name of PCR product	Forward 5′-3′	Reverse 5′-3′	Product size (bp)
p38α^D176A+F327S^ 5′End	bGHF2-TCCAGCCCGACCTCCCCTGGCACAACG	hs4R-GGCATTAAAGCAGCGTATCC	292
p38α^D176A+F327S^ 3′End	hs4F2-ATTGGGAAGACAATAGCAGGCATGC	oIMR9103-TCAAAGAGCAGCGAGAAGCGTTCAG	229
Pax7-Cre WT	1443 CTC CTC CAC ATT CCT TGC TC	1444 CGG CCT TCT TCT AGG TTC TG	496
Pax7-Cre mutant	1084 GCG GTC TGG CAG TAA AAA CTA TC	TT1885 GTG AAA CAG CAT TGC TGT CAC	100
R26-211	r26211 for TTGCCTCAAGAGGGGCGTGCTGAGCCAG	r26211 rev AGGACAACGCCCACACACCAGGTTAGCC	378

Pax7, paired box protein 7.

#### Induced expression of p38α^D176A+F327S^

Pax7-Cre/p38α^D176A+F327S^ mice were provided with regular diet or a diet supplemented with 625 mg/kg of dox in the chow [TD.01306 Rodent Diet (2018, 625 DOX), Envigo].

##### Anesthesia

Mice were injected with intraperitoneally overdose of 0.75 mg/ml ketamine + 0.1 mg/ml medetomidine/zsaline cocktail (purchased from the animal pharmacy in MD2, NUS) 0.015 ml/g followed by cardiac puncture. Tissues were then collected, processed, and/or stored for the analysis as detailed below.

#### Cell culture

C2C12 myoblast cell line was obtained from the ATCC. Cells were maintained in 10% FBS complete Dulbecco’s modified Eagle’s medium (Biological Industries) and incubated 37 °C and 5% CO_2_. To induce the myoblast to myotubes, process medium was changed to differentiation medium (97).

MG132 was purchased from MERCK (C2211-5MG).

##### Protein lysate preparations

Protein extracts of mice organs were prepared from liquid nitrogen snap-frozen organs in 2X tissue volume lysis buffer (50 mM Hepes pH = 7.5, 150 mM NaCl, 1 mM EDTA, 1% Triton X-100, 0.1% sodium deoxycholate, 0.1% SDS) that also contained HALT Protease and Phosphatase Inhibitors Cocktail (1009) (#78440; Thermo Fisher Scientific). Tissues were homogenized using Bullet Blender Tissue Homogenizer with the relevant beads (1X tissue volume - Next Advance) according to manufactures' instructions. Supernatant was collected and mixed with 2X SDS sample buffer, and boiled for 10 min.

Protein extracts of C2C12 cells were prepared by collecting cells in 2X SDS sample buffer after washing with PBS, and boiling the samples for 10 min.

Protein quantification was performed with MN Protein Quantification Assay (250) Reagents Kit (740967.250 MACHEREY-NAGAL, Duren).

### Immunoblotting

Thirty micrograms of proteins' samples were separated using 12% SDS/PAGE and transferred to a PVDF membrane (Bio-Rad) using Trans-Blot Turbo System (Bio-Rad). Membranes were blocked with 5% BSA in TBST (1X PBS with 0.1% Tween) for 60 min, then incubated with a primary antibody (diluted in 5% BSA in TBST) for 15 h, washed and incubated with a HRP-conjugated secondary antibody (diluted in 5% nonfat dry milk in TBST) for 90 min. Signal was developed using Western BrightTM ECL Kit (K-12045-D50 Advantsa) or Maximum Sensitivity Substrate Kit (34096 Thermo Fisher Scientific). Signal was detected using ChemiDOCTM MS Imaging System instrument (Bio-Rad Laboratories).

#### Primary mAbs

anti-HA (clone, 3F10) (11-867-423001; Roche), anti-GAPDH (Ab8245; Abcam). Primary polyclonal antibodies: anti-p38α (C20) (SC-535-G; Santa Cruz). Anti-phos-p38 (#4511), anti-MAPKAPK-2 (MK2) (#3042), anti-phos-MAPKAPK-2 (phos-MK2) (#3041) anti-MK3 (D54E4) (#7421), anti-MKK6 (D31D1) (#8550), anti-MKK3 (#5674), anti-Phospho-HSP27 (D1H2F6) (#9709), anti-p21 Waf1/Cip1 (#64016S), anti-p53(D2H90) (#32532), anti-Phospho-Histone H2A.X (Ser139) (#9718S), anti-Phospho-Histone H2A.X (Ser139/Tyr142) (#5438S), anti-cleaved caspase-3 (#9664), and anti-ubiquitin (E4I2J) (#43124) were purchased from Cell Signaling technology. HSP27 (AHO1132, Thermo Fisher Scientific). DKK3 (7O2I10, Thermo Fisher scientific). HRP-conjugated secondary antibodies: anti-rat (SC-2006) and anti-goat (SC-2354) were purchased from Santa Cruz, and anti-rabbit (#7074) and anti-mouse (#7076) from Cell Signaling technology.

### Histological analysis

Fresh mouse organs were fixed in 10 ml of 10% formalin (Sigma, HT501128-4L) for 24 to 48 h at room temperature (RT), followed by washing with PBS and preservation in 70% ethanol. Samples were submitted to Advanced Molecular Pathology Laboratory at the Agency for Science, Technology and Research (A∗STAR) in Singapore for preparation of paraffin blocks, 4 μm sections slides, and H&E staining. Imaging was performed using TissueFaxs for slide scanning. Skeletal muscle cross sections were pathologically evaluated for regeneration or degeneration process using the following ordinal scoring; 0 = nil, 1 = minimal, 2 = mild, 3 = moderate, 4 = marked, 5 = severe by Advanced Molecular Pathology Laboratory at the Agency for Science, Technology and Research (A∗STAR) in Singapore. Specifically, analysis was performed by a professional pathologist in a double-blind approach (he received the samples with no experimental information). Degeneration was evaluated by the size of deformed areas, while regeneration by the percentage of cells with central nuclei.

### Immunohistochemistry

Paraffin-embedded skeletal muscle sections were deparaffinized by soaking in Xylene for 10 min, following by rehydration in graded ethanol (100%, 95%, 90%, 70%, 50%) and then in DDW. Antigen retrieval was performed using pH 9.0 EDTA buffer, and 20 min in 2100 Antigen Retriever device according to the manufacturer’s instructions. Samples were then washed with PBS and blocked for 1 h at RT in blocking buffer (PBS containing 0.05% Tween-20, 0.3% Triton X-100 and 4% normal donkey serum). Primary antibodies in blocking buffer were incubated for 15 h at 4 °C in a humidity box. Fluorescent secondary antibody in blocking buffer were incubated for 2 h at RT and then mounted on slides using Immuno-mount (Thermo Fisher Scientific TA030FM). Slides were left for ∼15 h at RT to dry before stored at 4 °C. Imaging was performed by confocal microscopy (Olympus FV3000 Confocal Microscope). Primary antibody for laminin was purchased from Sigma (#L0663) and secondary fluorescent antibody was goat anti-rabbit IgG H&L (Alexa Fluor 488) (Abcam, ab150081).

### Fiber caliber calculation

Fibers area was evaluated as described in reference (98). Shortly, images of laminin-stained GC skeletal muscle cross sections were acquired using confocal microscopy (Olympus FV3000 Confocal Microscope). Morphometric analysis on digitized images of GC muscle sections was performed by implementing MuscleJ (an open-source image processing package based on ImageJ; imagej.net/plugins/cross-sectional-analyzer), as automated image analysis workflow in Fiji (imagej.net/software/fuju/downloads) (NIH). The percentage of frequency distribution of the fibers' size output was then calculated using GraphPad (www.graphpad.com).

### mRNA extraction

mRNA was extracted from snap-frozen mice skeletal muscle tissues using Bullet Blender Tissue Homogenizer with stainless steel beads (Blend 0.9–2.0 mm) (Next Advance - NA SSB14B) in PureZOL RNA Isolation Reagent (Bio-Rad, #7326890). For C2C12 cells, cells were collected with PureZOL RNA Isolation Reagent (Bio-Rad, #7326890). Aurum Total RNA Fatty and Fibrous Tissue Module (Bio-Rad, #7326870) was used for RNA purification, according to the manufacturer’s protocol.

### Quantitative real-time PCR

iScript genomic DNA Clear cDNA Synthesis Kit (#172-5035, Bio-Rad) was used for cDNA synthesis (1 μg RNA each reaction) according to the manufacturer’s instructions. Samples were then mixed with iTaq Universal SYBR Green Supermix (#172-5121, Bio-Rad) and run with 7500 Fast Real-Time PCR (Applied Biosystems, model 4351106) for RT-PCR quantification. Results were normalized to β2 microglobulin for C2C12 cells and to 18S rRNA for mice skeletal muscle tissues. Analysis was performed using 2-Δ Ct approach.

Primers used for quantitative real-time PCR are shown in Table 2.

**Table 2:** Primers used for qRT-PCR

Name of gene	Forward 5′-3′	Reverse 5′-3′
P38α	GAGAAGATGCTCGTTTTGGAC	GGACTGGTCATAAGGGTCAGC
P38α^D176A+F327S^ (human)	GCCGAGCTGTTGACTGGAAG	GGAGGTCCCTGCTTTCAAAGG
MKK6	GCCGCCTCGGGATTTAGA	CACCTCAAAGTTCTGGTTTCCAA
MKK3	TGGACTCCCGGACCTTCA	GTCATCAGCCTCCACTTCGAA
MK2	ACCGAGGAGACCAGGCATT	CGCCGATGCTCTTCATGAT
MK3	CCCACCGAGATGTCAAGCC	GGGTGTCTGGAGGGCATTT
HSPB1 (HSP27)	ATCCCCTGAGGGCACACTTA	GGAATGGTGATCTCCGCTGAC
Pax7	TCTCCAAGATTCTGTGCCGAT	CGGGGTTCTCTCTCTTATACTCC
Cdkn2a (P16INK4a)	CGCAGGTTCTTGGTCACTGT	TGTTCACGAAAGCCAGAGCG
Cdkn1a (p21)	CCTGGTGATGTCCGACCTG	CCATGAGCGCATCGCAATC
18S rRNA	GTAACCCGTTGAACCCCATT	CCATCCAATCGGTAGTAGCG
β2 Microglobulin	TTCTGGTGCTTGTCTCACTGA	CAGTATGTTCGGCTTCCCATTC

Pax7, paired box protein 7; qRT-PCR, quantitative real-time PCR.

RNA-seq analysis, detection of differentially expressed genes, and k-mean clustering of DE genes were performed as previously described (41, 99, 100, 101).

### Program versions for analysis

**Table:** 

FastQC:	v0.11.9, http://www.bioinformatics.babraham.ac.uk/projects/fastqc/
cutadapt:	v2.10, http://cutadapt.readthedocs.org/en/stable/
fastq_quality_filter:	v0.0.14, FASTX package, http://hannonlab.cshl.edu/fastx_toolkit/
TopHat:	v2.1.1
full command:	tophat -G genes.gtf -N 6 --read-gap-length 5 --read-edit-dist 11 --segment-length 20 --read-realign-edit-dist 3 --no-coverage-search --library-type fr-first strand genome processed.fastq.gz
htseq-count:	v0.6.0, http://www-huber.embl.de/users/anders/HTSeq/doc/count.html
DESeq2:	v1.26.0
R:	R version 3.6.1, with packages RColorBrewer_1.1-2, pheatmap_1.0.12, ggplot2_3.2.0 and ggrepel_0.8.1

### Enrichment analysis

#### IPA

The group of “KMupdown” consists of the genes that were changed in mice treated with dox-supplemented diet compared to mice that were fed with regular diet for 2 months in two independent experiments (K-n = 2) (M-n = 3).” This group of genes was submitted to enrichment analysis using IPA (QIAGEN Inc., https://www.qiagenbioinformatics.com/products/ingenuity-pathway-analysis/). A canonical pathway is considered as significant when the BH *p* value was below 0.05 (or −logBH *p* value larger than 1.3). Activation Z-score is considered as significant when larger than two or smaller than −2.

### Statistical analysis

Statistical significance of the differences between groups was determined by ordinary one-way ANOVA or unpaired two-tailed *t* test with the GraphPad Prism software (∗*p* < 0.05, ∗∗*p* < 0.01, ∗∗∗*p* < 0.001, and ∗∗∗∗*p* < 0.0001). Note that this software fully supports *post hoc* tests after performing an ANOVA. All data are expressed as mean ± SEM.

## Data availability

Data are to be shared upon request. Contact information: David Engelberg (engelber@mail.huji.ac.il).

## Supporting information

This article contains supporting information.

## Conflict of interest

The authors declare that they have no conflicts of interest with the contents of this article.
