# Supplementary material for: Chronic activation of p38α in skeletal muscle causes necrotic changes, but also abolishes expression of MK2, MK3, and MKK6 and the muscle recovers
Source: J Biol Chem. 2026 Mar 4;302(4):111338. doi: 10.1016/j.jbc.2026.111338 (PMC13066812; doi:10.1016/j.jbc.2026.111338)
Supplement: Supplementary Material — 1 [file mmc1.docx]

Legends to supplementary files

File S1. List of the molecules that compose the clusters shown in Figure 3D.

File S2. List of enriched pathways and the molecules modified in these pathways in the experiments of this study.
