# Supplementary material for: Chronic activation of p38α in skeletal muscle causes necrotic changes, but also abolishes expression of MK2, MK3, and MKK6 and the muscle recovers
Source: J Biol Chem. 2026 Mar 4;302(4):111338. doi: 10.1016/j.jbc.2026.111338 (PMC13066812; doi:10.1016/j.jbc.2026.111338)
Supplement: Supplementary Material 4 [file mmc4.pdf]

Figure S1

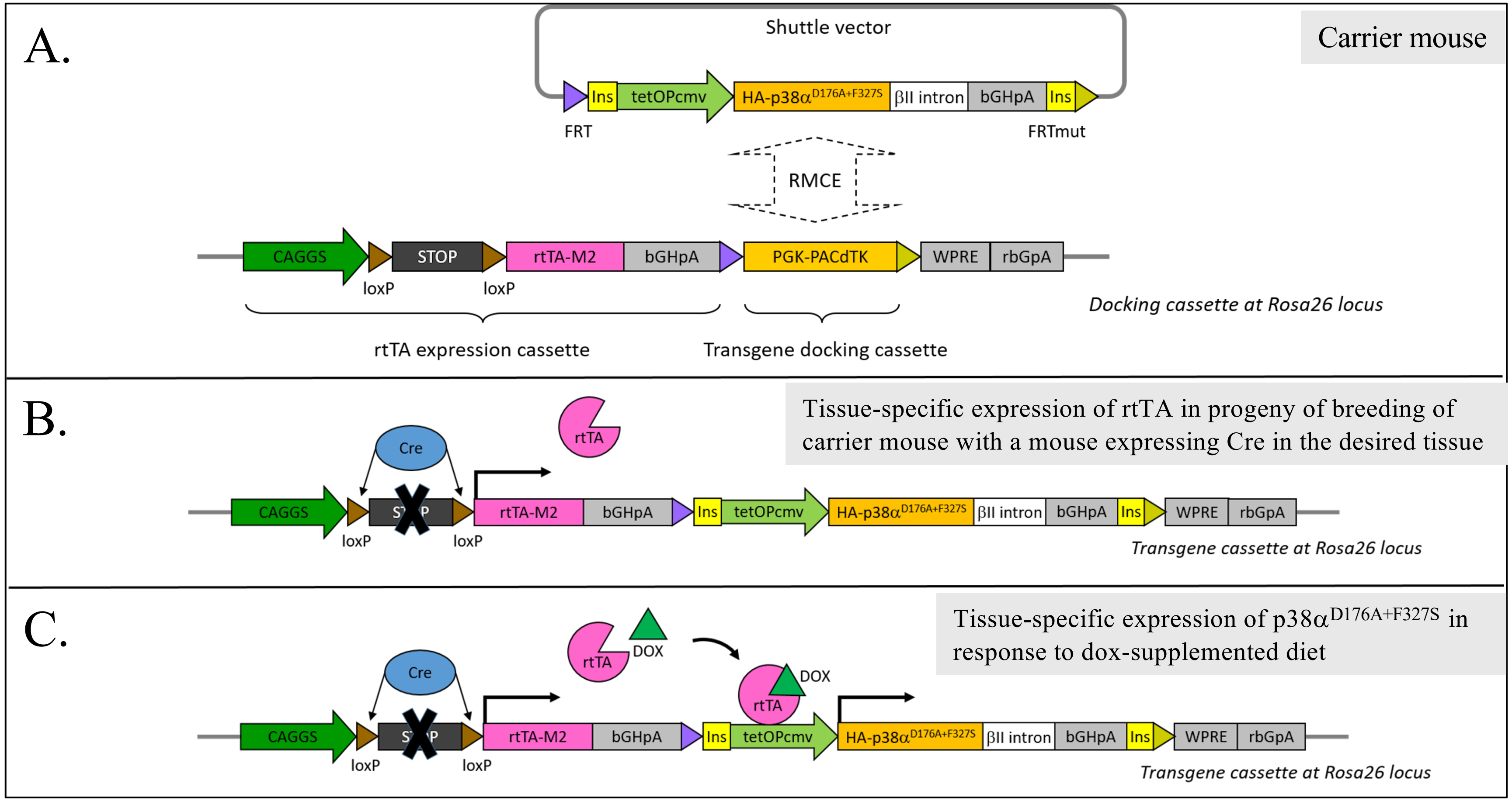

Figure S1. Schematic description of the transgenic mouse model used in this study (the figure is adopted, largely, from reference (51). A) The two expression cassettes inserted in the genome of the 'carrier' mice: 1) an 'rtTA expression cassette' (bottom part of panel A). 2) a 'transgene docking cassette'. The cassettes were integrated into mouse ES cells at the Rosa26 locus via homologous recombination. Transcription of rtTA-M2 is driven by the strong and constitutively active CAGGS promoter, but intervening sequence (STOP) prevents transcription. The STOP sequence is bordered by flox sequences so that the rtTA gene would be transcribed only when the 'STOP' is removed. The 'transgene docking cassette' contains a PGK promoter driving hybrid selection marker (PACdTK) allowing for both positive and negative selection by puromycin and ganciclovir, respectively. The upper part of panel A shows the shuttle vector which contains the transgene expression cassette, comprised of the HA-tagged p38α$^{D176A+F327S}$ coding sequence driven by the tet-responsive promoter (tetOPcmv), a splicing acceptor/donor sequence (βII intron) and a polyadenylation signal sequence (bGHpA). The entire transgene expression cassette is flanked by an insulator sequence (Ins) and a FRT and FRT mutant (F3) sequence. The p38α$^{D176A+F327S}$ expression cassette is integrated into the transgene docking region by highly efficient RMCE process. Note that the tetOP-CMV promoter would be activated only if rtTA is expressed and is bound todoxycycline. **B)** The expression system as appears in cells and tissues expressing the cre-recombinase, for example in skeletal muscle of Pax7-Cre/p38a$^{D176A+F327S}$ mice. The 'STOP' sequence is removed by cre-recombinase (shown in blue) and as a result rtTA is transcribed and consequently translated (shown in pink). **C)** The expression system as appears in cells and tissues expressing cre (in this study in skeletal muscle) after dox-supplemented diet is provided (dox is shown in green triangles). rtTA-dox complex is transcriptionally active, binds to the tet responsive promoter and the p38α$^{D176A+F327S}$ transgene is expressed.
